# Benefits and concerns of seeking and experiencing lucid dreams: benefits are tied to successful induction and dream control

**DOI:** 10.1093/sleepadvances/zpac027

**Published:** 2022-09-07

**Authors:** Remington Mallett, Laura Sowin, Rachel Raider, Karen R Konkoly, Ken A Paller

**Affiliations:** Department of Psychology, Northwestern University, Evanston, IL, USA; Department of Biology, Massachusetts Institute of Technology, Cambridge, MA, USA; Department of Psychiatry, University of Rochester Medical Center, Rochester, NY, USA; Department of Psychology, Northwestern University, Evanston, IL, USA; Department of Psychology, Northwestern University, Evanston, IL, USA

**Keywords:** sleep, dreams, lucidity, nightmares, emotion, Reddit, content analysis

## Abstract

Therapies focused on lucid dreaming could be useful for treating various sleep disorders and other conditions. Still, one major roadblock is the paucity of systematic information on the consequences of attempting these sorts of dreams. The current study sought to quantify positive and negative aspects of seeking lucid dreams, describe their phenomenology in detail, and identify features associated with positive or negative experiences. Observational data from a massive lucid-dream discussion forum were analyzed to capture lucid-dreaming themes. Forum posts were independently rated on multiple dimensions hypothesized to contribute to the valence of lucidity-related phenomena. Our results revealed that lucid dreams can end nightmares and prevent their recurrence, but they can also induce harrowing dysphoric dreams. The realization of dreaming (lucidity) and dreams with high-control were both associated with positive experiences. We translated our results into a process model that describes the progression from lucid dream induction to waking benefit, identifying potential areas of concern. Our results and model suggest that negative outcomes primarily result from failed induction attempts or lucid dreams with low dream control, and that successfully inducing high-control lucid dreams poses low risk for negative outcomes. Lucid dreaming has valuable therapeutic and recreational potential, but a better understanding of the risks is required. Our findings provide new insights into possible negative repercussions and how to avoid them in future applications.

**Statement of Significance**

The unpredictability of dream content hinders scientific progress and leads to suffering in nightmare patients. Controlling dream content through lucid-dreaming has been hailed as a solution to these problems, though it is increasingly clear that this approach is not without limitations. This study identified and characterized benefits and concerns of seeking lucid dreams by utilizing social media. Negative experiences, such as sleep paralysis and restlessness, were associated with failed lucid-dreaming attempts rather than lucid dreaming itself. Positive experiences, such as enhancing dream content and mood benefits, were associated with high amounts of dream control. These findings define the terrain of potential outcomes of lucid-dreaming practice and illuminate ways to promote positive outcomes in future applications.

## Introduction

Dreams in which one is aware that a dream is in progress—lucid dreams [1]—hold promise as a recreational and therapeutic tool. Lucid dreams have been described since antiquity [2] but are relatively rare in contemporary society [3]. They can be induced using a variety of approaches [4] in research, clinical, and home settings. Much remains to be learned about how lucid-dreaming can impact sleep and mental health, and there are various potential downsides that could negate the benefits [5, 6]. Given the increasingly common use of these practices, it is important to characterize these risks and delineate strategies through which they can be minimized.

Most prior work on lucid dreaming has focused on its upsides. Lucid dreams are generally more positive than non-lucid dreams [7–11], potentially as a consequence of the dream control that commonly co-occurs with lucidity [12]. Survey studies consistently reveal that the primary motivation and objective for most lucid dreamers is to have fun [13, 14], for example, choosing to fly or have sex during the dream [15, 16]. The positivity of lucid dreaming has led to the use of lucid-dreaming therapy to reduce nightmare frequency [17, 18] and alleviate insomnia symptoms [19]. Other benefits of lucid dreaming with preliminary empirical support include skill rehearsal [20], creative inspiration [21], and personal growth [22].

What are the downsides to the practices engaged by those seeking to have a lucid dream? Whereas most concerns are anecdotal, there is strong empirical support for the occasional occurrence of lucid nightmares. In a subset of lucid dreams, the dreamer is unable to alleviate a terrifying dream experience through intentional control or deliberate awakening [23–25]. Sleep paralysis is another negative experience [26, 27] with a strong association to lucidity [26, 28, 29], though it does not involve dream awareness and the direction of this relationship is unclear. Other than negative dream experiences themselves, the use of some lucid-dream-induction methods might disrupt sleep [6] and increase dissociative symptoms [5, 30, 31]. Disrupted or irregular sleep patterns are associated with lucid dreaming [32, 33], though many recent studies have not found negative impacts of lucid dreaming on more general sleep quality [10, 34–37]. Still, it seems difficult to avoid the notion that sleep disruption is explicitly required for some induction methods [4, 38].

The goal of the current study was to characterize the full spectrum of benefits and concerns of attempts to engage in lucid dreaming. We conducted a content analysis using social media posts from a popular online lucid-dreaming forum, allowing us to identify benefits and concerns surrounding lucid dreaming with less bias than traditional survey studies [39]. Our findings offer a comprehensive account of what can go right, what can go wrong, and what should be emphasized in future applications of lucid dreaming.

## Methods

In brief, we ran a content analysis on posts from a public lucid dreaming internet forum to develop positive and negative themes, and then used theme counts along with additional manual ratings for statistical comparisons. This research was deemed exempt from IRB review by the ethics committee of Northwestern University due to the use of publicly available and anonymous data. The ethics of internet and social media research is an evolving conversation [40–44] and we followed recommended community guidelines [42]. As an additional precaution, all examples presented in the manuscript are paraphrased from direct quotes [41].

### Data source

**Figure 1.**
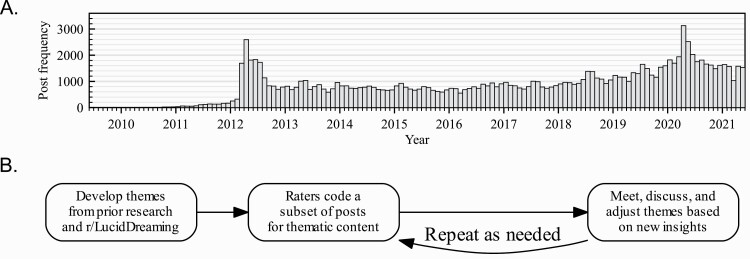
Dataset and methods. (A) The lucid-dreaming subreddit (r/LucidDreaming) activity over time. r/LucidDreaming became active in late 2010 and has remained active with roughly 1000 posts per month. Note that a subset of 400 posts was extracted for the current analyses. (B) Deductive/inductive content analysis procedure. Three coders developed an initial set of positive and negative themes based on prior lucid-dreaming literature and a cursory glance at r/LucidDreaming, and continued to adjust themes as the analysis proceeded.

Reddit is one of the most visited websites globally and is frequently used for psychological studies [41, 45]. The self-proclaimed “front page of the internet”, Reddit is an anonymous discussion forum where users can create and self-moderate sub reddits dedicated to specific topics of interest [46]. For example, on the chess sub reddit, users are only allowed to post content related to chess. Each sub-reddit consists of topically relevant posts, where users attach comments to discuss the post. We analyzed posts from the most active subreddit focusing on the topic of lucid dreaming^1^ (hereafter r/LucidDreaming), which had approximately 419 000 members as of October 28th, 2021. The average monthly post rate was 943 from January 2015 to December 2019 (Figure 1A). Though we don’t have demographic information for r/LucidDreaming users, the general Reddit user demographics from a 2016 PEW study^2^ are 67% male, 64% between the ages of 18 and 29, 70% white non-hispanic, and 82% with some college education (see also [45]).

### Data collection

Public Reddit data is viewable and available for access through a variety of sources. We extracted all r/LucidDreaming posts from the Pushshift dataset [47] using the PSAW Python package.^3^ For the current analysis, we extracted a subset of 400 posts (first 200 in April 2019 and first 200 in July 2019). Reddit posts were analyzed—as opposed to comments—as posts are more representative of the topics discussed on a given sub reddit. Importantly, there is far more topical content on r/LucidDreaming than we were interested in for the current investigation into positive and negative themes (e.g. lucid dream-induction methods, requests for help with lucid dreaming, scientific articles, videos, etc.). All 400 posts were coded, and before resolving coding disagreements, 213 of them contained >1 of our themes as identified by >1 coder. After resolving all coding disagreements, 174 posts remained as containing >1 theme identified by all coders. This remaining sample size of 174 posts is consistent with prior Reddit content analyses [48] and was included in all further analyses.

### Data analysis

Three authors (R.M., L.S., R.R.) performed all stages of a content analysis [49, 50]. Following prior related work [48], we used a combination of deductive (top-down) and inductive (bottom-up) approaches to the theme development stage of analysis [49] (Figure 1B). We also placed a valence restriction on theme development by only including themes that had an inherent positive or negative sentiment. Thus, posts were not coded for valence explicitly, but were coded for each of the themes that had either positive or negative sentiment. Note that while each theme itself is valenced in the broad sense, each post contributing to a theme did not necessarily match the valence. For example, a post about a neutral sleep paralysis experience would still fit under the “negative” sleep paralysis theme, since in general sleep paralysis is viewed as a frightening, unwanted, and negative experience [27].

We developed an initial codebook of specific coding guidelines and initial themes for each coder to use for analysis. Posts were coded in batches and then all coders would reconvene to resolve minor discrepancies and make adjustments to the codebook if necessary [49, 51]. At intermediate stages of coding, we calculated inter rater reliability for each theme as the percentage of all posts where all raters were in complete agreement. Ultimately, any of the posts where there was not complete agreement were resolved through group discussion (thus, final percent agreement was 100%). Posts were coded using Doccano^4^ and aggregated for summary statistics using Python. The final themes, along with a brief description and example for each theme, are presented in Table 1. Posts could include more than one theme, though we used a subset of single-valence posts to compare the frequency of positive-only (containing >1 positive themes and zero negative themes) and negative-only (>1 negative themes and zero positive themes) posts using a proportion *z*-test.

**Table 1.** Abbreviated final themes and guidebook for content analysis

	Theme	Brief definition
Positive	Creativity/insight	Any explicit reference to a creative action in the dream or a realization made while dreaming that has an outcome on waking life
	Dream enhancement	Any reference to dreams being enhanced by lucidity, including but not limited to pleasure-seeking, excitement, and vividness
	Nightmare resolution	Any time an explicit connection is made between lucid-dreaming and the reduction of bad dream content
	Positive waking mood	Any reference to lucidity resulting in a positive mood during the day, especially upon awakening in the morning
	Rehearsal	Any reference to the use of lucid dreams as a virtual practice ground for a waking activity
Negative	Lucid dysphoria	Any reference to a dysphoric/bad dream or nightmare where the dreamer is lucid and unable to do anything positive about it
	Poor sleep	Any reference to poor or degraded sleep quality, including that as a result of induction methods and poor sleep leading to lucidity
	Reality confusion	Any expression of being wrong about one’s state of consciousness (excluding typical non-lucid dream misperceptions and sleep paralysis). This includes wake being dream-like and false awakenings
	Sleep paralysis	Any reference to sleep paralysis or related phenomena (e.g. visual or auditory hallucinations, a feeling of weight on the chest)
	Unwanted lucid dreams	Any mention of a user having naturally occurring lucid dreams and not being able to make them stop despite wanting to

To investigate any difference in popularity between positive and negative themes, we analyzed theme frequency in relation to up-votes and comments. For each theme, we identified the median up-vote ratio (up- to down-votes) of posts and the median number of comments attached to posts. Each of up-vote ratio and comment frequency were compared between positive and negative themes with a Mann–Whitney *U* test using the Pingouin Python package [52].

Based on inductions from the main content analysis, we analyzed what factors distinguish positive-only from negative-only posts. For this analysis, positive-only and negative-only posts were stripped of all prior coding assignments, shuffled in order, and coded along two dimensions by two authors (L.S., R.R.). Posts were coded as to whether they involved a lucid dream experience of any kind (yes/no) and the presence of dream control (high/little-to-none). Degrees of dream control can be defined in various ways, though we simplified our analysis by defining high dream control as “the ability to successfully execute an intention to its intended outcome” (c.f. [12, 53]). Posts that did not include a dream experience at all, or had no identifiable level of control were removed from this analysis. Both of these factors were tested for a relationship with the valence of a lucidity-related theme (positive/negative) with a chi-square test using the Pingouin Python package [52].

## Results

**Figure 2.**
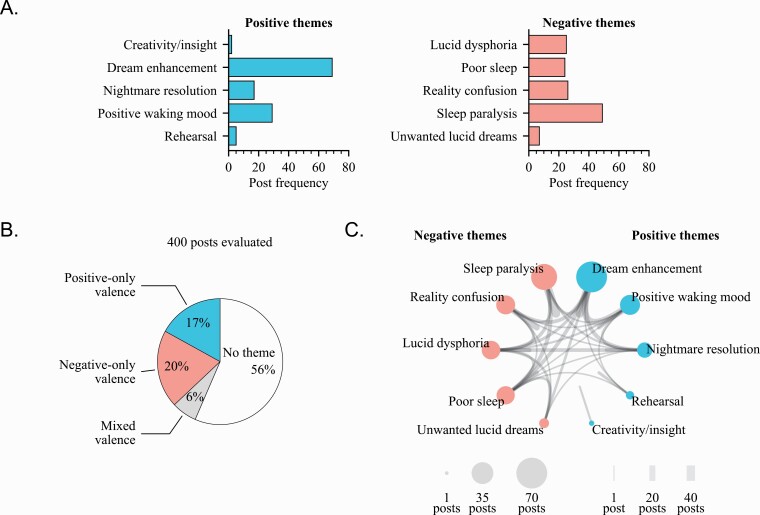
Total theme counts. (A) The total number of posts that were identified for each positive and negative theme. (B) Relative post frequencies aggregated by valence and in relation to the total sample. Posts could include multiple themes. (C) Theme overlap. In this network visualization, outer nodes are themes and sized proportionally to their post count, and edges represent the amount of posts that included both themes. Unconnected edges indicate the amount of posts that included only that theme.

Final positive and negative lucid-related themes are presented in Table 1. Of the 400 reviewed posts, 174 unique posts were identified as including one or more of our ten themes (from 166 unique users, max 4 posts for a single user). A theme was identified 253 times across the 174 posts, with 115 posts including one theme, 41 posts including two themes, 16 posts including three themes, and 2 posts including four themes. Of the original 400 posts, 24% (94 posts from 93 unique users) were identified as including at least one positive theme and 26% (106 posts from 99 unique users) were identified as including at least one negative theme. Total counts for individual themes are presented in text below and in Figure 2A, with representative examples presented in Tables 2 and 3. Overlap between themes is presented in Figure 2C. One-hundred forty-eight posts were coded with only one valence (>1 positive and 0 negative, or vice versa), where the frequency of positive-only (68) and negative-only posts (80) were statistically similar (*z* = 0.99, *p* = .32; Figure 2B). Positive and negative themes did not differ in post popularity when operationalized as upvote ratio (*p* = .82) nor comment frequency (*p* = .52).

**Table 2.** Paraphrased positive theme examples

Positive theme	Representative post excerpts
Creativity/insight	I’d like to try and play with optical illusions and impossible objects while constructing things in my lucid dreams
Dream enhancement	Becoming lucid for the first time was just as amazing as I had hoped I spent years doing all kinds of weird stuff in my lucid dreams. Things like flying and changing myself into different creatures Just came out of a vivid lucid dream where I was completely aware of being asleep. There was such a feeling of freedom After realizing it was just a dream, I took control and did anything I desired In my lucid dreams, I like to just look around. It’s amazing what my mind is capable of. There is so much detail and it’s all so realistic My only motivation to train myself to lucid dream is so I can have sex with my favorite movie character Became lucid last night. I was pretty happy about it, since lucid dream sex is gratifying
Nightmare resolution	Became lucid during a nightmare and intentionally woke myself up. During the dream I knew that just waking up my real self could help to end the terrible dream As soon as I knew I was dreaming, I looked them in the eyes and told them to go away I recognized that it was all just a dream, so I opened my eyes and got out Over time, I learned to recognize when I was having a nightmare. I would wake myself up, it felt like my real body was moving. I’m glad because now I never have nightmares I always knew when I was dreaming back when I was younger. If I wanted to leave the nightmare, or any dream, I just pushed on my head and I’d always wake up
Positive waking mood	I woke up happy from my lucid dream and excited about future dreams I had a lucid dream! How exhilarating. It’s also a good sign for my lucid training Finally!!! Just had my first lucid dream, it was so cool After more than a week, I had a lucid dream and did not wake up immediately. Yes!! I’m looking forward to more. So thankful for my mind
Rehearsal	Can I use lucid dreams to advance my technical skills? I imagined playing my favorite video game with lots of detail and accuracy

**Table 3.** Paraphrased negative theme examples

Negative theme	Representative post excerpts
Lucid dysphoria	My lucid dreams become nightmares, can anyone help!? I get a chill down my spine whenever I recognize the dream. It’s scary Once lucid, it’s like the harder I attempt to change the dream, the wilder they become. Sometimes they change into nightmares, like the time I tried to influence the dream but instead the zombie apocalypse began One time, while lucid, I wanted to know what death would feel like. At some point, a nuclear blast hit. My vision stopped and I was awash in a horrible smell from the other dead people. I started to suffocate but could not wake up
Poor sleep	I have daily vivid lucid dreams that are so intense I feel exhausted when I wake up. I am more restless in the morning than I was before I went to sleep the night before After waking up for MILD, I just stayed awake despite efforts to become lucid After a while, all the lucidity becomes tiring and I just want to relax
Reality confusion	I just perform a reality check whenever I wake up because I get false awakenings a lot I had a long and scary series of false awakening recently. I repeatedly woke up and noticed something dream-like to trigger lucidity I feel like I looped for more than an hour, but eventually I woke up for real But I didn’t really wake up. I had only awoke in my dream At this point, I struggle to determine whether I’m dreaming or awake. It’s bad and making me feel strange
Sleep paralysis	Scared and immobilized. Not even able to scream. So then I figure I’m likely in sleep paralysis But as soon as I am close to sleeping, an odd sound makes me think something is crawling towards me There are still demons around me during sleep paralysis, but they don’t bother me anymore I won’t try to lucid dream just because I’m nervous about getting sleep paralysis When I try to MILD I hear strange noises, like a ringing
Unwanted lucid dreams	How can I stop lucid-dreaming?? They’re great, but sometimes it’s too much Once I was lucid-dreaming long enough that I became bored and just wanted to have a non-lucid dream. I woke myself up but fell back into another lucid dream. Eventually I lost lucidity, but it took a bit too long

### Positive themes

The most common positive theme was dream enhancement, with positive waking mood and nightmare resolution roughly similar in second. Themes creativity/insight and rehearsal were barely present at all on r/LucidDreaming. Total counts and representative examples of positive themes are presented in Figure 2A and Table 2, respectively.

#### Creativity/insight

 We identified very few posts (2 posts from 2 unique users) that included reference to creativity, insight, or problem solving (inter rater reliability = 99%, median up-vote ratio = 1, median comment frequency = 2). Neither of these posts described specific experiences where a lucid dream was used for creativity, rather the post questioned the possibility of using them for a creative endeavor. One post was a question about the possibility of finding lost items in a lucid dream, and the other was a comment about wanting to build “complex figures” while lucid dreaming.

#### Dream enhancement

Dream enhancement was the most common of all 10 themes on r/LucidDreaming with a total of 69 posts (from 69 unique users; interrater reliability = 77%, median upvote ratio = 3, median comment frequency = 2). Posts in this category overwhelmingly described specific accounts of enhanced dreams, with very few posts dedicated to questions or comments. Representative posts were those in which users described lucid dreaming being “as cool as they imagined”. Flying and sex were common activities that were either described (e.g. “lucid dream sex is not just fun, but rewarding”) or expressed as something to be desired (e.g. “the reason I’m trying to lucid dream is for sex”). Many dream enhancement posts included some level of dream control to take specific actions (e.g. “I was able to fly just by asking”). In a large subset of posts, dreamers did not not change the dream environment, though displayed at least normal dream control—deliberate control over their mentation and behavior while dreaming, but not items in the environment [53]. This was occasionally used for active inspection of the dream scene. For example, one user described how while lucid they would “intentionally look around at what their mind was capable of, astounded by the detail and realism”. Rather than changing the dream, lucid dreams with normal control were enhanced by an amazement and wonder about the vividness of the dream, only possible with the critical reflection of lucidity. Other dreams involved not even bodily dream control, but instead seemed to have a positive emotion coincide with the moment of lucidity (e.g. experiencing an “overwhelming feeling of liberation”). Thus, the enhancement of dreams with lucidity seems to exist to some degree at varying levels of dream control.

#### Nightmare resolution

There were 17 unique users who wrote 17 posts that included reference to lucid dreams being utilized to overcome a dysphoric dream situation (interrater reliability = 93%, median upvote ratio = 2, median comment frequency = 1). Posts in this theme were split primarily between detailed accounts of overcoming a specific nightmare with lucidity and a more general description of using lucid dreaming as a regular practice to overcome nightmares, especially early in life. In the examples of targeted nightmare resolution, the dreamer would either change the content and stay in the dream (e.g. “once I became lucid I told them to leave”) or end the dream completely to wake up (e.g. “the nightmare ended after I woke myself up”). Both seem effective at ending negative dream content. Many users described how they used lucid dreaming to overcome their nightmares during childhood or adolescence. One user explained how they would “get out of weird dream situations as a child”. Importantly, users reported that using lucidity to reduce nightmares has a lasting impact, as in the following example: “After developing a habit of becoming lucid and waking myself up from nightmares, I don’t have any at all”.

#### Positive waking mood

There were 29 posts from 28 unique users that included a positive waking mood sentiment (inter rater reliability = 88%, median up-vote ratio = 7, median comment frequency = 3). Positive waking mood seemed to follow either a particularly positive dream or one’s first lucid experience that occurred after a series of failed induction attempts. Posts expressed a general elation or excitement upon awakening, as in: “So hype! It was more astonishing than anything else I’ve experienced”. Similar to the nightmare resolution posts, on occasion the positive waking mood seemed to have lasting impacts. For example, one user noted that after lucidity, they “woke up happy and became excited about dreaming”, while another user began to “gain confidence” after achieving lucidity.

#### Rehearsal

There were five unique posts from five unique users referencing lucid dreams as any kind of rehearsal space (inter rater reliability = 98%, median up-vote ratio = 1, median comment frequency = 8). Only one post referenced an explicit instance of rehearsing during a lucid dream, and it was a user who, while dreaming, “imagined playing their favorite video game”. The other four posts were requests for information about the possibility of using lucid dreams for rehearsal or learning in some way (e.g. “Can I use lucid dreams to advance my technical skills?”).

### Negative themes

The most common negative theme on r/LucidDreaming was sleep paralysis by far, followed by lucid dysphoria, poor sleep, and reality confusion themes, which all had similar counts. Total counts and representative examples of negative themes are presented in Figure 2A and Table 3, respectively.

#### Lucid dysphoria

There were 25 posts from 25 unique users related to dysphoric lucid dream content (inter rater reliability = 83%, median up-vote ratio = 2, median comment frequency = 3). These posts all expressed very negative dream experiences where the dreamer was aware of the dream and unable to use their lucidity to get out of the situation, thus fitting the criteria of a lucid nightmare [25]. Posts mostly described a dysphoric dream experience with lucidity but no dream control, as in the following example: “Once lucid, my attempts to control the dream failed and monsters showed up. It was painful”. Notably, lucidity was sometimes more simply associated with feelings of terror, as in the case where a user recognized they were dreaming only to “become anxious and curl up”. Another user claimed, “whenever I get lucid, the first thing that happens is panic and despair”. Other posts described a regular occurrence of lucid dysphoria (e.g. “My attempts at control always make the dream worse, even nightmares”), and often with the user asking for advice from others (e.g. “Can anyone help me stop my lucid dreams from becoming nightmares?!”).

#### Poor sleep

There were 24 posts from 24 unique users related to poor sleep (inter rater reliability = 84%, median up-vote ratio = 2, median comment frequency = 3). These posts equally mentioned lucid dreaming increasing poor sleep and poor sleep prefacing a lucid dream. Lucid dreaming led to poor sleep through two routes. In some cases, the induction methods used to deliberately induce a lucid dream resulted in reduced sleep for a night (e.g. “After waking up for MILD, I just stayed awake despite efforts to become lucid.”). In other cases, poor sleep, or waking up feeling less rested, resulted from the intensity of the lucid dream itself. These posts were generally from high-frequency and natural lucid dreamers, as in the following example: “I have daily vivid lucid dreams that are so intense I feel exhausted when I wake up”. Such users would frequently request tips on how to prevent lucid dreams (see unwanted lucid dreams theme). Notably, not all poor sleep posts were negative, and in many cases referred to how poor sleep likely led to a lucid dream. Some users became lucid during a nap following some form of sleep deprivation.

#### Reality confusion

There were 26 posts from 26 unique users related to dream/reality confusion (inter rater reliability = 88%, median up-vote ratio = 2, median comment frequency = 2.5). The majority of these posts were instances of false awakenings (a dream of waking up), and a smaller subset referenced a concern of having dream-like feelings during waking. False awakening posts were typically emotionally neutral with the user simply stating they had a false awakening, though a subset were negative (e.g. “I hate false awakenings”). Dream-reality confusion would happen when a user could no longer tell if they were dreaming or not. There were also rare instances of a single experience that combined false awakenings with dream-reality confusion, as in a long series of false awakenings leading to waking dream-reality confusion: “after a bunch of false awakenings I had to do a reverse reality check to check if I was awake”.

#### Sleep paralysis

The highest negative theme count was for the sleep paralysis theme, with 49 posts in total (from 44 unique users; inter rater reliability = 81%, median up-vote ratio = 2, median comment frequency = 4). Most sleep paralysis experiences included explicit references to a negative feeling (e.g. “It was scary, I couldn’t yell, couldn’t move”), though in some cases users were not alarmed or concerned with the sleep paralysis, and in a few cases even enjoyed it (e.g. “had sleep paralysis but it wasn’t scary”). Many sleep paralysis posts were related to the use of lucid-dream-induction methods, some of which aim to encourage and harness sleep paralysis to achieve lucidity [4, 54]. In particular, the mnemonic induction method (MILD) commonly co-occurred with sleep paralysis phenomena (e.g. “When I try to MILD I hear strange noises, like a ringing”). Expectation was a critical feature of sleep paralysis posts. For one user, “I won’t try to lucid dream just because I’m nervous about getting sleep paralysis”, and for others the knowledge about sleep paralysis prior to the event eliminated the typical fear association (e.g. “there are still demons around me during sleep paralysis, but they don’t bother me anymore”).

#### Unwanted lucid dreams

There were 7 posts from 7 unique users related to unwanted lucid dreams (inter rater reliability = 97%, median up-vote ratio = 1, median comment frequency = 2). Most of these posts were explicit requests for feedback about how to stop having lucid dreams, seemingly from natural lucid dreamers (e.g. “How can I stop lucid dreaming??”). In these posts, users often expressed that their lucid dreams were very vivid and cognitively active to the point of being exhausting and impeding normal sleep, as in the following example: “After a while, all the lucidity becomes tiring and I just want to relax”. Notably, this was the only theme for which we found no prior literature.

### Contributors to post valence

Inter rater reliability was high for both lucidity attainment (91%) and dream control (80%) codings. The following results were qualitatively similar when either set of codings was used, so one was selected for presentation arbitrarily (L.S.).

#### Attaining lucidity

**Figure 3.**
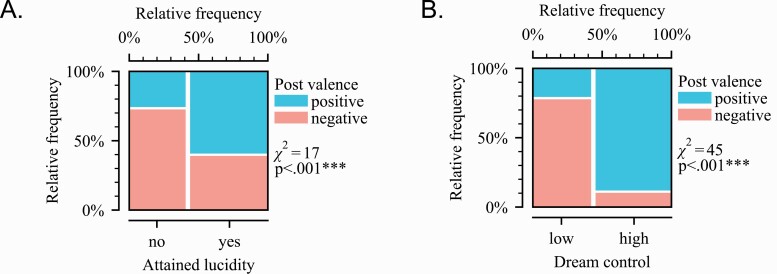
Contributions to post valence. (A) The relationship between the presence of a lucid dream and post/theme valence. A post was more likely to be positive if it included a lucid dream experience. Each of the four mosaic plot tiles are sized proportionally to the number of observations. A greater size difference between tiles indicates a stronger relationship between lucidity and valence. (B) The relationship between dream control and post/theme valence. A dream experience was more likely to be positive if it involved a high level of dream control. A greater size difference between tiles indicates a stronger relationship between lucidity and dream control.

There was a significant relationship between attaining lucidity and the valence of a post, *X*^2^ (1, *N* = 148) = 17.4, *p* < .0001 (Figure 3A). Posts that included a lucid dream experience were more likely to be positive than those without.

#### Dream control

There was a significant relationship between the presence of dream control and the valence of a dream experience, *X*^2^ (1, *N* = 90) = 44.7, *p* < .0001 (Figure 3B). Posts including a dream experience were more likely to be positive if they had a high amount of dream control, relative to little-to-no dream control.

## Discussion

Social media posts provided a wealth of information about lucid-dream-related experiences. Reddit users came to r/LucidDreaming to discuss not only their lucid dream experiences, but also induction techniques and other associated phenomena. They described personal experiences from the recent and distant past, asked questions about possibilities and fears, and requested or offered advice. Many users wanted to know if others had shared similar experiences as their own.

### Benefits

Dream enhancement was the most prevalent theme among r/LucidDreaming posts, consistent with nearly all prior survey studies of lucid dreaming. Dreams were enhanced via lucidity in a variety of ways. We observed many accounts of enhanced dreams stemming from increased vividness and deliberate dream control. Many posts described the use (or desired use) of dream control to promote flying or sex [16]. Other positive uses of dream control were unique and personalized, such as performing magic spells, traveling to specific locations [8], or recreating events from a favorite video game.

In addition, dreams were enhanced despite lacking deliberate dream control. Some users would, upon lucidity, start to “actively look” or investigate the lucid dream to appreciate “how much detail” and “how realistic” it was. Here, a sense of awe was instilled from the observation of a vivid perceptual dream experience while knowing they were asleep. Some reported intense positive dream emotions that coincided directly with lucidity (e.g. a “feeling of freedom” upon the realization of dreaming). Together, these low-control lucid dreams suggest that full control over the dream environment is not a necessary precursor to the positive emotions that co-occur with lucidity [7–11]. In at least some cases, positive emotion might arise from observing the vividness of the dream first-hand. These and related “clear light” lucid dreams [55, 56] suggest that people derive benefit from witnessing their dream content, even in the absence of control. An interesting future direction would be to perform an in-depth analysis on posts with the dream enhancement theme. It’s possible that there are variable strengths of enhancement across these different approaches.

Many posts referenced a positive waking mood, suggesting that the aforementioned dream enhancements translate to a more positive waking life [14, 22, 57–60]. Positive waking mood posts primarily referred to the mood immediately following a lucid dream, and the phenomenology of these accounts offers insight into what might drive a similar effect in empirical studies [1, 10]. For example, many posts contained overlapping themes of positive waking mood and dream enhancement, suggesting that dream enhancements led to enhanced morning-mood. These posts included some form of exhilaration or excitement upon awakening after controlling or witnessing the dream. Thus, one plausible mechanism behind a morning-mood benefit of lucidity is a positive emotion carry-over from the dream into waking [61]. By comparison, the induction of a sense of awe during waking can promote positive emotions and prosocial behaviors [62], so a similar experience during lucid dreaming—or a memory of the dream [63]—might inspire the same [60]. Another possibility is that a positive mood results from a sense of achievement when a lucid dream ultimately occurs after a prolonged series of failed attempts.

Another benefit identified was the reduction of nightmares with lucid dreaming. This finding adds to mounting subjective reports and case studies of successful nightmare reduction through the use of lucid dreaming [18]. Notably, users reported self-prescribing lucid-dreaming therapy. Though nightmares are common, they are often left untreated due to a lack of emphasis in sleep medicine [64]. Some users described lasting impacts from the use of lucid dreaming, even experiencing no nightmares for years (see also [65]).

### Concerns

The frequent topic of sleep paralysis was unsurprising given its prior association with lucid-dreaming frequency [26, 28, 29]. Sleep paralysis is typically viewed as a negative and unwanted experience, and we observed this perspective often, including users who had never experienced it but were afraid it might occur. However, we also found that—in the context of lucid-dreaming practices—the experience of sleep paralysis was occasionally sought out rather than feared, mostly as a vehicle to induce lucidity. Some posts even referred to sleep paralysis as a pleasurable experience and offered advice on overcoming potentially unnecessary fears.

Reality confusion occurs in many forms, and in the context of dream research often refers to source confusion over whether a memory was originally experienced while asleep or awake [66]. However, we observed very few source confusions. Most reality confusion could be traced back to instances of false awakenings, which are instances where someone incorrectly believes they’ve woken up, only to later realize the awakening was dreamt. False awakenings have shown prior positive associations with lucid-dreaming frequency and sleep paralysis [29, 67]. In the current study, isolated incidences of single-series false awakenings were largely benign, and occasionally even used to promote lucid dreaming. In contrast, long-series false awakenings—where a user was repeatedly wrong about waking up—occasionally digressed into dream-reality confusion while awake (see anoneirognosis [68]). The current dataset suggests this effect might only last a single morning, though future work could help identify to what degree reality confusion after false awakenings persists into waking life.

Our results offer accounts of sleepless nights after failed (and sometimes frustrating) induction attempts. Notably, the sleep loss here was rarely reported as persistent over time, but instead was linked to lucid-dream-induction methods. Similarly, not all accounts of sleep loss were described as negative, since it occurred prior to successful induction attempts. Users also reported spontaneous lucid dreams occurring after a natural bout of disrupted sleep, consistent with prior work suggesting that fragmented sleep might lead to a lucid dream [69, 70]. Sleep loss due to induction methods is consistent with prior concerns and a motivating factor for improving induction methods via alternate approaches.

Another topic in posts about poor sleep was that lucid dreams may interfere with the restorative role of sleep, given that they might be more active than non-lucid dreams [71]. One user claimed they would “wake up more tired than before the sleep started”, and another claimed their lucid dreams “were exhausting”. These few posts came almost exclusively from natural and high-frequency lucid dreamers who were unable to avoid becoming lucid. Sleep with lucid dreams might result in the perception of reduced restorative sleep for high-frequency lucid dreamers because of the dream awareness, as they suggest. Alternatively, the unique neural features of lucid REM sleep [72, 73] might interfere with some REM sleep functions. Another idea is that sleep fragmentation surrounding the lucid dream [69, 70] might promote subjective tiredness. Of course this possible concern would not be relevant for those who lucid dream infrequently, but it raises the intriguing possibility that there may be a need for approaches to reduce lucid dream frequency. Future work might prospectively investigate whether lucidity prevents some restorative role of sleep and dreaming, as there is little empirical support for this relationship currently [10, 34–37].

Our observation of lucid dysphoria extends recent reports of lucid nightmares [24, 25] by suggesting that—while small in numbers—such experiences can be very frightening and should not be dismissed. Users offered accounts of terrifying lucid dreams that they were unable to control, despite strenuous efforts in some cases. It might be that the increased vividness and heightened emotions often associated with positive lucid dreams also apply to these negative experiences. The lack of dream control was the most consistent feature of lucid dysphoria, suggesting that effective methods to increase dream control would be valuable.

### Distinguishing positive and negative themes

We identified two features that distinguish positive from negative lucid-related phenomena. The attainment of a lucid dream and having high dream control were associated with positive experiences. This finding has important implications for applications of lucid dreaming, because by identifying specific features that separate positive from negative experiences, the process can be biased away from valid concerns and towards benefits. Generally speaking, the goal of a lucid-dreaming practice is to go from deliberate induction to positive benefit, but the intermediate steps and mechanisms are largely uncharted. We translated our results into a process model that begins to address this shortcoming.

**Figure 4.**
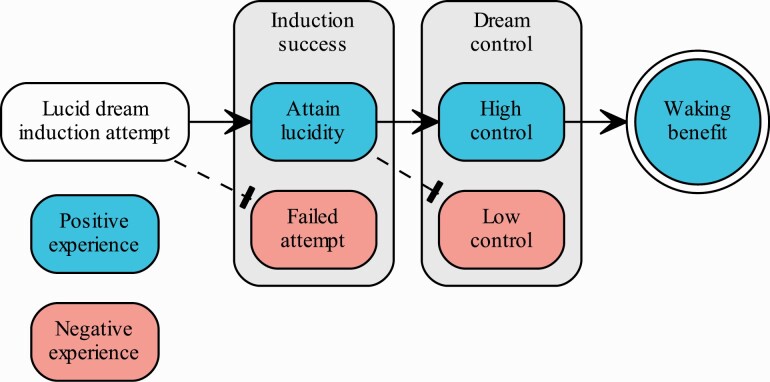
Process model for lucid-dreaming benefits. Between a lucid-dream-induction attempt and its waking benefit are two critical branch points (surrounding boxes). The lucid-dream inductee either continues with positive experiences towards the waking benefit (solid arrows) or the process ends in a negative experience (dashed arrows).

Our model of lucid-dreaming benefits (Figure 4) is centered around two branch points which determine a person’s success in having positive experiences. The first point occurs during the induction attempt. Based on our content analysis and findings from Figure 3A, a rule emerges where the inductee either becomes lucid or does not. If lucidity is attained, the experience is likely to be positive and the process continues towards a waking benefit. If lucidity is not attained, the process ends, likely with a negative outcome such as poor sleep or sleep paralysis. The second point occurs after lucidity is attained. Findings presented in Figure 3B suggest that the rule here is based on the amount of control. The inductee either has high/sufficient control or does not. With sufficient dream control, a positive outcome such as nightmare resolution or dream enhancement is highly likely. Without such control, the process is likely to end in a negative experience such as lucid dysphoria.

In summary, our model proposes at least two critical checkpoints—induction success and degree of dream control—that lie between an induction attempt and its potential waking benefit. We described these stages as a linear process, though the temporal relationship between dream awareness (i.e. lucidity) and control is not clear [74]. Another simplification in our model is that we treated lucidity, control, and emotion all as binary, when they more likely fall along a continuum [1]. We expect a simple variant of this model accounting for degrees of these features to make similar predictions. Thus, the findings from this study and the resulting process model suggest that the successful induction of a high-control lucid dream warrants low concern for negative outcomes and supports reaching a positive outcome.

### Limitations

Reddit users are not representative of the general population [45]. However, most psychology studies—including those investigating lucid dreaming—other systematic biases, primarily towards undergraduate student samples [75]. For example, the majority of articles within a recent meta-analysis of lucid-dreaming frequency focused on student samples [3]. With Reddit being used by nationalities and age groups of all kinds, our sample likely contains a wider demographic span than prior research.

While we do not have access to r/LucidDreaming demographics specifically, we can speculate that they follow general Reddit demographics. If so, the bias towards young, white, middle-class males would be consistent with our observation of minimal creativity/insight posts, since the use of lucidity for creative purposes is more commonly reported by older females [13]. It is also possible that Reddit contains a larger proportion of gamers than other social media platforms. It has been suggested that lucid dreaming and specifically dream control increase with video game use [76, 77] (though see [78]), and if r/LucidDreaming follows this trend it might have influenced our theme totals (e.g. inflating dream enhancement and deflating lucid dysphoria).

Another influence on our sample is what motivates someone to post on Reddit. For example, since r/LucidDreaming is a popular place for anyone to ask questions about lucid dreaming, this might have inflated positive or negative themes (though the similar amount of up-votes and comments between themes suggests this did not play a major role). It’s also possible that users were intentionally lying (trolling) for other reasons [79], although online anonymity has historically been viewed as a shield for privacy and is thought to promote overall honest reporting in research settings [80].

The lucid-dreaming sub reddit contains many more discussions of important themes that were not part of the current focus, which was limited to only positive or negative valenced themes. Just over half of all coded posts did not include any of our valenced themes. We did not analyze these posts extensively, but we note that they sometimes contained relevant videos or articles yet mostly focused on lucid-dream-induction methods. People often posted to offer or request assistance in successfully inducing lucid dreams. In the scientific literature, the successful induction of lucid dreams is also a critical focus and is to-date largely unresolved [4, 81]. Though laboratory technology is sometimes involved [82], many cognitive lucid-dream-induction methods can be utilized at home with minimal prior experience. Thus, r/LucidDreaming might contain insights into novel approaches as well as the efficacy of popular existing approaches. Additionally, the frequent requests for help provide evidence of a societal interest in becoming aware during dreams. Future work utilizing this dataset to investigate other topics—especially lucid-dream-induction methods—would likely be a fruitful path.

## Conclusion

We obtained descriptive accounts of emotionally charged themes prevalent in the lucid-dreaming community. The positive themes observed support prior accounts of lucid dreaming used in and outside clinical settings. The negative themes signal potential risks, but our results suggest they are limited to specific and identifiable instances that could be minimized if not entirely avoided in controlled settings (e.g. by efficiently inducing high-control lucid dreams). Our findings show that these rich experiential accounts provide direction for future efforts to amplify the benefits of lucid dreaming and lower the risks.

## Footnotes

https://www.reddit.com/r/LucidDreaming.

http://pewrsr.ch/21jq0bC.

https://github.com/dmarx/psaw.

https://github.com/doccano/doccano.

## Availability of materials

Data and code are available in a public repository: https://osf.io/bce8y
